# Supplementary material for: Prognostic and Predictive Significance of Stromal Tumor-Infiltrating Lymphocytes (sTILs) in ER-Positive/HER2−Negative Postmenopausal Breast Cancer Patients
Source: Cancers (Basel). 2022 Oct 4;14(19):4844. doi: 10.3390/cancers14194844 (PMC9564297; doi:10.3390/cancers14194844)
Supplement: Supplementary file 1 [file cancers-14-04844-s001.zip › cancers-1902807-supplementary.pdf]

Supplementary Table S1. TILs scores in association with clinicopathological variables among all the patients.

**Variables**	**Low TILs <10% n (%)**	**Intermediate TILs 10-39% n (%)**	**High TILs ≥40% n (%)**	**P-value**
Tumor size	N=744			**<0.001**
≤20mm	494 (83)	53 (9)	48 (8)	
>20mm	95 (64)	15 (10)	39 (26)	
Tumor grade (NHG)	N=614			**<0.001**
1	110 (96)	5 (4)	0 (0)	
2	302 (86)	27 (8)	22 (6)	
3	68 (46)	26 (18)	54 (36)	
ER	N=732			**<0.001**
Negative	78 (49)	28 (17)	54 (34)	
Positive	497 (87)	41 (7)	34 (6)	
PR	N=622			**<0.001**
Negative	202 (67)	36 (12)	64 (21)	
Positive	281 (88)	24 (8)	15 (5)	
HER2	N=635			**<0.0001**

Negative	461 (82)	52 (9)	47 (8)	
Positive	31 (41)	11 (15)	33 (44)	
Ki67	N=543			**<0.0001**
<15%	335 (88)	26 (7)	21 (5)	
≥15%	85 (53)	27 (17)	49 (30)	
IHC Subtypes	N=626			**<0.0001**
ER+/HER2-	406 (89)	32 (7)	20 (4)	
HER2+	31 (42)	11 (15)	32 (43)	
TNBC	48 (51)	20 (21)	26 (28)	
PAM50	N=519			**<0.0001**
Basal	15 (31)	12 (25)	21 (44)	
Luminal B	89 (81)	8 (7)	13 (12)	
HER2-enriched	22 (42)	8 (15)	22 (42)	
Luminal A	240 (92)	15 (6)	6 (2)	
Normal	42 (88)	4 (8)	2 (4)	
70-gene signature	N=519			**<0.0001**

Ultra-low risk	79 (100)	0 (0)	0 (0)	
Low risk	197 (91)	13 (6)	6 (3)	
High risk	132 (59)	34 (15)	58 (26)	
CD4	N=520			0.22
Low	200 (81)	20 (8)	27 (11)	
High	209 (77)	27 (10)	37 (14)	
CD8	N=520			**<0.0001**
Low	235 (92)	14 (5)	7 (3)	
High	174 (66)	33 (13)	57 (22)	
CD19	N=520			**<0.0001**
Low	243 (96)	8 (3)	3 (1)	
High	166 (62)	39 (15)	61 (23)	
FOXP3	N=520			0.24
Low	204 (81)	23 (9)	26 (10)	
High	205 (77)	24 (9)	38 (14)	
IMMUNE 1	N=519			**<0.0001**
Low	294 (88)	19 (6)	20 (6)	

High	114 (61)	28 (15)	44 (24)	
IMMUNE 2	N=519			**<0.0001**
Low	318 (90)	14 (4)	6 (2)	
High	90 (50)	33 (18)	58 (32)	

In bold, statistically significative P values

Supplementary Table S2. Univariate and multivariable Cox regression analysis of DRFI. as an endpoint to assess the prognostic value of TILs and other clinical variables in HER2+ and TNBC subtypes

**TILs**	**Univariate DRFI[a]**<br>**HR (95% CI)**	**Multivariable DRFI[b]**<br>**HR (95% CI)**
HER2+	(n=37)	(n=55)
<10% (Ref)	1.00	1.00
10-39%	1.03 (0.26-4.14)	0.59 (0.12-2.99)
≥40%	1.18 (0.38-3.65)	0.38 (0.08-1.77)
TNBC	(n=48)	(n=69)
<10% (Ref)	1.00	1.00
10-39%	0.46 (0.10-2.17)	1.55 (0.53-4.53)
≥40%	0.51 (0.14-1.96)	1.40 (0.45-4.37)

. [a] univariate analysis including untreated patients (control). [b] multivariable analysis adjusting for tumor size, tumor grade, Ki67, ER, PR, HER2, and TAM (all subtypes) or adjusted for tumor size, tumor grade, Ki67, and TAM (HER2+ and TNBC). (Ref) Reference

Supplementary Table S3. Univariate and multivariable Cox regression analysis of BCSS as an endpoint to assess the prognostic value of TILs in HER2+ and TNBC subtypes.

TILs	Univariate BCSS [a] HR (95% CI)	Multivariable BCSS [b] HR (95% CI)
HER2+	(n=37)	(n=55)
<10% (ref)	1.00	1.00
10-39%	1.33 (0.32-5.58)	0.69 (0.11-4.36)
≥40%	1.40 (0.43-4.61)	0.32 (0.05-1.88)
TNBC	(n=48)	(n=69)
<10 %(ref)	1.00	1.00
10-39%	0.23 (0.03-1.87)	1.36 (0.43-4.25)
≥40%	0.36 (0.08-1.71)	1.34 (0.42-4.27)

[a] univariate analysis including untreated patients (control). [b] multivariable analysis adjusting for tumor size, tumor grade, Ki67, ER, PR, HER2, and TAM (all subtypes) or adjusted for tumor size, tumor grade, Ki67, and TAM (HER2+ and TNBC). (Ref) Reference.

Supplementary Table S4. Univariate Cox regression analysis of DRFI as an endpoint to assess the prognostic value of different TILs markers and two immune gene modules among HER2+ and TNBC untreated patients.

	**CD8[a]**	**CD4**	**FOXP3**	**CD19**	**IMMUNE 1**	**IMMUNE 2**	**PD1**	**PD-L1**
	Univariate DRFI HR (95% CI)							
HER2+ (n=31)								
Low	1.00	1.00	1.00	1.00	1.00	1.00	1.00	1.00
High	0.99 (0.34-2.88)	0.80 (0.28-2.29)	0.98 (0.34-2.82)	0.87 (0.24-3.14)	0.55 (0.17-1.78)	0.81 (0.23-2.92)	0.83 (0.23-2.99)	**0.36 (0.13-1.04)**
TNBC (n=39)								
Low	1.00	1.00	1.00	1.00	1.00	1.00	1.00	1.00
High	0.62 (0.15-2.46)	1.34 (0.36-4.99)	0.88 (0.24-3.29)	0.80 (0.20-3.18)	**0.25 (0.06-1.02)**	1.93 (0.24-15.47)	0.42 (0.11-1.55)	1.35 (0.28-6.49)

[a]CD8 Low=q1-3, CD8 High= q4 otherwise the median was used as cut-off. In bold, statistically significative, or borderline hazard ratios.

Supplementary Table S5. Multivariable Cox regression analysis of DRFI as an endpoint to assess the prognostic value of different TILs markers and two immune gene modules among HER2+ and TNBC untreated patients.

	CD8[a]	CD4	FOXP3	CD19	IMMUNE 1	IMMUNE 2	PD1	PD-L1
	Multivariable DRFI[b] HR (95% CI)							
HER2+ (n=55)								
Low	1.00	1.00	1.00	1.00	1.00	1.00	1.00	1.00
High	0.89 (0.32-2.46)	0.44 (0.16-1.23)	0.47 (0.17-1.27)	0.84 (0.25-2.89)	0.40 (0.13-1.16)	0.87 (0.27-2.75)	0.49 (0.18-1.34)	0.85 (0.26-2.78)
TNBC (n=74)								
Low	1.00	1.00	1.00	1.00	1.00	1.00	1.00	1.00
High	0.92 (0.39-2.16)	2.19 (0.91-5.30)	1.04 (0.44-2.46)	2.40 (0.68-8.44)	**0.42 (0.17-1.01)**	**8.94 (1.07-75.07)**	1.90 (0.63-5.72)	**3.15 (0.91-10.89)**

[a]CD8 Low=q1-3, CD8 High= q4 otherwise the median was used as cut-off. [b] multivariable analysis adjusting for tumor size, tumor grade, Ki67, and TAM (HER2+ and TNBC). In bold, statistically significative, or borderline Hazard Ratios.

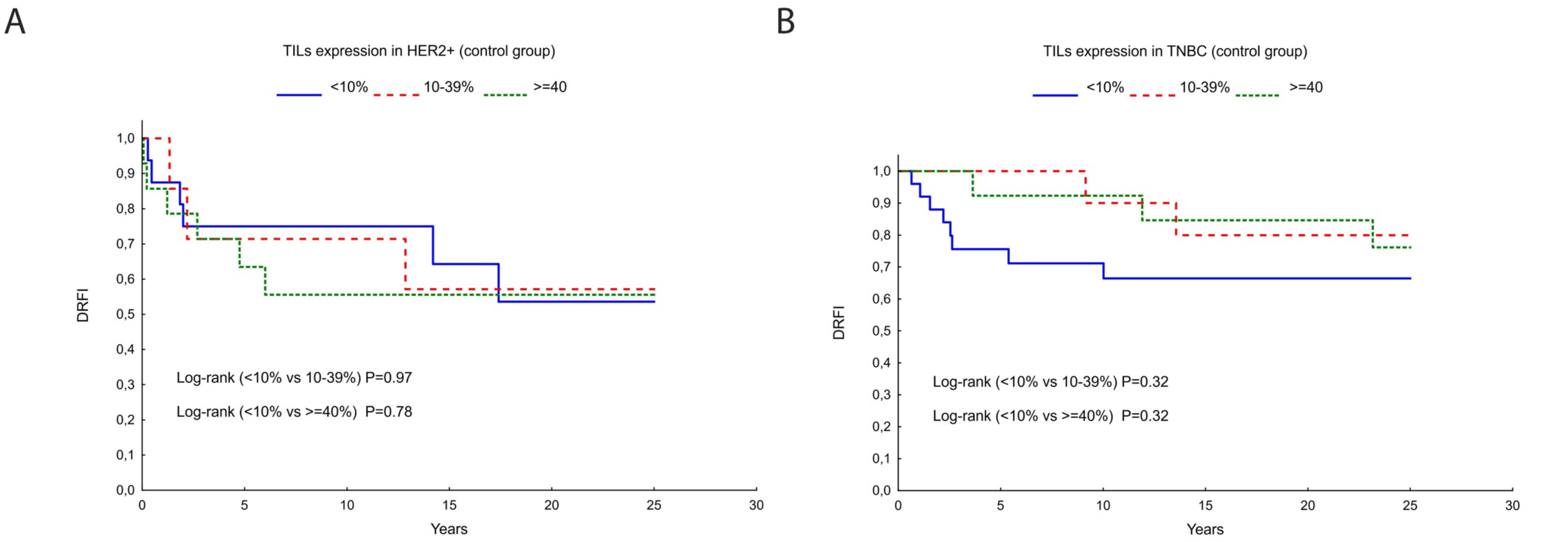

No at risk

TILs <10%	16	12	10	6	5	4
TILs 10-39%	7	5	5	4	3	2
TILs >=40%	14	8	7	7	6	4

No at risk

TILs <10%	25	17	15	13	12	5
TILs 10-39%	10	10	9	8	7	4
TILs >=40%	13	12	12	11	10	8

Supplementary Figure S1. Kaplan-Meier survival analysis showing TILs prognostic value within the control group regarding the endpoint distant recurrence-free interval (DRFI) in (**A**) HER2+ and (**B**) TNBC. Tumors were categorized into low (<10%), intermediate (10-39%) or high (≥40%) TILs. Risk tables are shown below the graphs and P values correspond to the Log-rank test
